# Optimized Aluminum Reflector for Enhancement of UVC Cathodoluminescence Based-AlGaN Materials with Carbon Nanotube Field Emitters

**DOI:** 10.3390/molecules26134025

**Published:** 2021-06-30

**Authors:** Manoj Kumar Chandra Mohan, Sang Kyun Shim, June Key Lee, Nakwon Jang, Naesung Lee, Wael Z. Tawfik

**Affiliations:** 1 Department of Materials Science and Engineering, Chonnam National University, Gwangju 61186, Korea; 177329@jnu.ac.kr (M.K.C.M.); sinboozon1@naver.com (S.K.S.); 2 SBK Materials Co., Gwangju 61186, Korea; 3 Division of Electrical & Electronic Engineering, Korea Maritime University, Busan 49112, Korea; nwjang@kmou.ac.kr; 4 Department of Nanotechnology and Advanced Materials Engineering, Sejong University, Seoul 05006, Korea; nslee@sejong.ac.kr; 5 Department of Physics, Faculty of Science, Beni-Suef University, Beni-Suef 62511, Egypt

**Keywords:** Al reflector, AlGaN materials, CNTs, UVC emission, cathodoluminescence

## Abstract

The far ultraviolet C (UVC) light sources based on carbon nanotube (CNT) field emitters as excitation sources have become promising light sources for sterilization, disinfection, and water purification. However, the low light extraction efficiency of UVC–CNT light sources still hinders the practical application of these structures. Herein, we report an optimized aluminum (Al) reflector to enhance the light extraction efficiency of UVC–CNT light sources. Optical analysis of UVC-CNT light sources covered by the Al reflectors with various thicknesses ranging from 30 to 150 nm was performed to realize the optimized reflector. The UVC-CNT light sources exhibit the highest light extraction efficiency when the Al reflector layer has an optimized thickness of 100 nm. For comparison, the cathodoluminescence (CL) spectra were recorded for UVC–CNT light sources with and without the optimized Al reflector. The measured light output power and the estimated power efficiency of the UVC–CNT light-source-tube with Al reflector were enhanced by about 27 times over the reference. This enhancement is mainly attributed to the outstanding reflection effect of the Al reflector.

## 1. Introduction

Ultraviolet C (UVC) light source tubes based on carbon nanotubes (CNTs) field emitters are promising to replace conventional UVC light-emitting diodes (LEDs) for applications related to air and water sterilization and decontamination [1,2,3]. UVC LEDs, which rely on the use of AlGaN nitride epitaxial layers, are still not efficient enough to be considered as possible alternatives on a large industrial scale [4,5,6]. This inefficiency is for different reasons and is related to the quality of the nitride material with high aluminum (Al) composition and/or the technology employed for fabricating the diodes, where low external quantum efficiency is observed. One of the issues concerns the deepening of the Mg-acceptor level, which would make it difficult to deposit the high-conductivity p-type layer [7,8,9,10]. In addition, the poor structural quality of the AlGaN epitaxial layers would cause dislocations and create non-radiative defects that destroy the internal quantum efficiency of the LEDs [11,12,13,14,15]. Furthermore, these devices currently display low light extraction efficiency and output power [16,17,18,19]. To address these issues, the electron beam pumped method was employed by several groups for the UVC–excitation from nitrides semiconductors. The device fabricated with this method, UVC light source tubes, has no requirement for a low-conductivity p-type layer, which is a unique advantage over the conventional UVC–LEDs [20,21,22,23]. However, to avoid the issues related to the conventional thermionic e-beam pumped method, such as the hot emission nature of metallic cathode and the high accelerating voltage, CNT field emission (FE) was utilized as the excitation source for electrons in UVC light sources [24,25,26]. Generally, in UVC light source devices based on CNTs–FE, the heat is not required to emit the electrons; rather, the emission of the electrons is controlled by the electric field. Moreover, the cold emission nature of CNTs–FE effectively prevents the thermal drift of the emitted electrons, allowing better and more stable electron emission over time with a high current density [27]. On the other hand, to improve the light extraction efficiency of the used AlGaN epitaxial structure as a target (anode) in these devices, numerous studies have employed the Al cap layer on the top surface of the AlGaN epitaxial layer [28]. Al is an excellent reflector with reflectivity of about ~0.92 in the whole UV spectrum range because of its low refractive index and high extinction coefficient [29]. However, none of these studies have considered the effects of the Al layer’s existence and thickness on the output of UV light source tubes. Given the importance of the Al reflector in optical devices based on nitride materials, the effect of the Al layer on extraction efficiency must be taken into account. In this work, AlGaN heterostructure-based UVC emission was grown on a conventional 2-inch sapphire substrate to serve as a CL layer in a light source tube with and without the Al reflector layer, to examine its effect on the extraction efficiency. To determine the optimum thickness of the Al layer, optical analysis was performed on a UVC-CNT light source tube with different Al layer thicknesses ranging from 30 nm to 150 nm. After that, the optimized Al reflector was applied in a high-quality AlGaN heterostructure. Then, the vacuum packaging UVC–CNTs light source tube was fabricated in a triode mode structure through the combination of a CNTs field emitter and an AlGaN heterostructure based UVC emission with and without optimum Al cap layer as a CL layer. 

Typically, triode mode structure consists of a CNTs cathode plate, an AlGaN-based anode, and a metal grid gate electrode of mesh type. The main functions of the gate in the light source tube is to decrease the value of the threshold voltage by reducing the spacer height between the cathode and anode, the gate is inserted between them, as well as to disperse the flight path of electrons emitted from the cathode to achieve uniform brightness at the anode surface. In this mode, the screen-printed CNTs are well aligned with the holes of the metal grid gate electrode to facilitate electron extraction from the cathode to the anode. The light extraction efficiency and output powers of these UVC–CNTs light source tubes were experimentally measured and compared. The results indicate that the UVC–CNTs light source tube-based AlGaN structure CL layer with the optimized reflector achieves light output power higher than that without the Al reflector and the optimized reflector can enhance the light extraction efficiency of UVC–CNTs light source tubes. This work is one step towards realizing a high-performance light extraction UVC–CNTs light source tube-based AlGaN materials for commercial applications.

## 2. Results and Discussion

**Figure 1:**
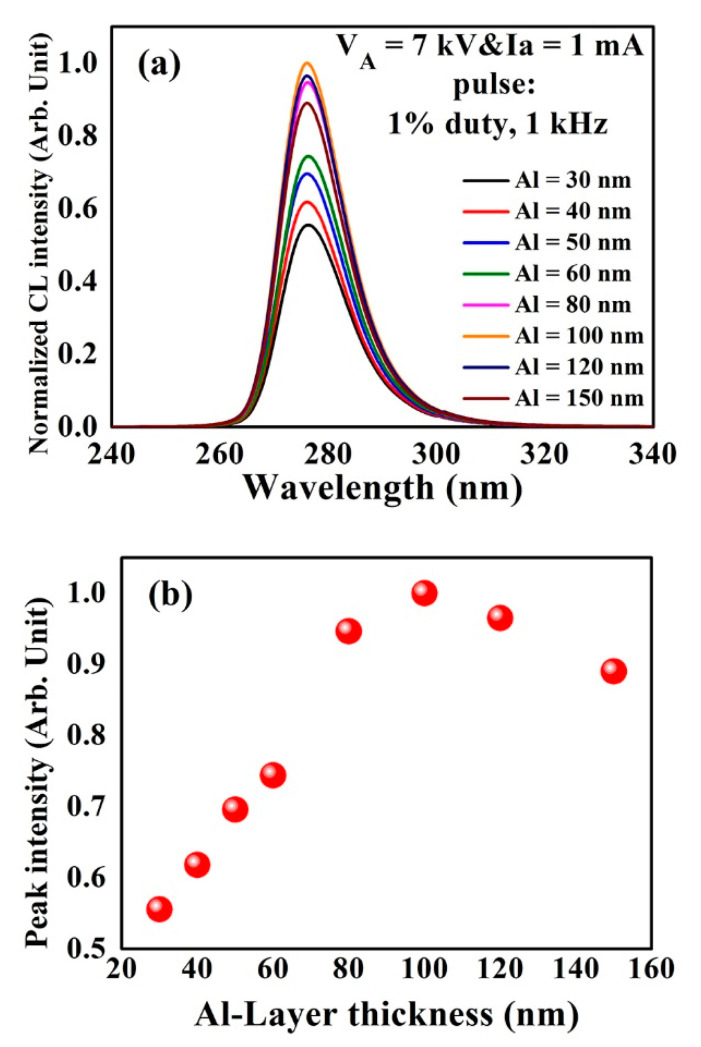
Optical analysis of the UVC–CNTs light source tube covered by the Al reflectors with different thicknesses ranging from 30 to 150 nm at a fixed V_A_ of 7 kV and I_A_ of 1 mA. (**a**) Normalized CL spectra; (**b**) peak intensity of the CL spectra in (**a**) with various thicknesses of Al reflector layer.

To achieve an optimized Al reflector, optical measurements were performed to analyze the effect of the reflector with different thicknesses on the light extraction efficiency of the UVC–CNTs light source tube. Figure 1a shows the measured CL emission spectra of AlGaN-based UVC structure covered by the Al reflectors with various thicknesses ranging (30 to 150) nm. When the thickness of the Al reflector increases from (30 to 150) nm, the CL emission intensity first increases, and then decreases. The AlGaN-based UVC structure covered by the Al reflector with a thickness of 100 nm achieved the highest CL emission intensity, as shown in Figure 1b. The results illustrate that the Al reflector with the optimized thickness of 100 nm can enhance the CL intensity of the AlGaN-based UVC structure, owing to the increase of the light extraction from the top side of the UVC-CNTs light source tube. As the thickness of the Al layer increased from (0 to 100) nm, the reflectivity was increased. After that thickness, the electron absorption in the Al layer was increased. An optimum Al layer thickness is achieved by trading off high reflectivity (for thick Al) against electron absorption losses, which would favor a thinner layer. In general, Al metal has a very high extinction coefficient and low refractive index, making it a good reflector.

**Figure 2:**
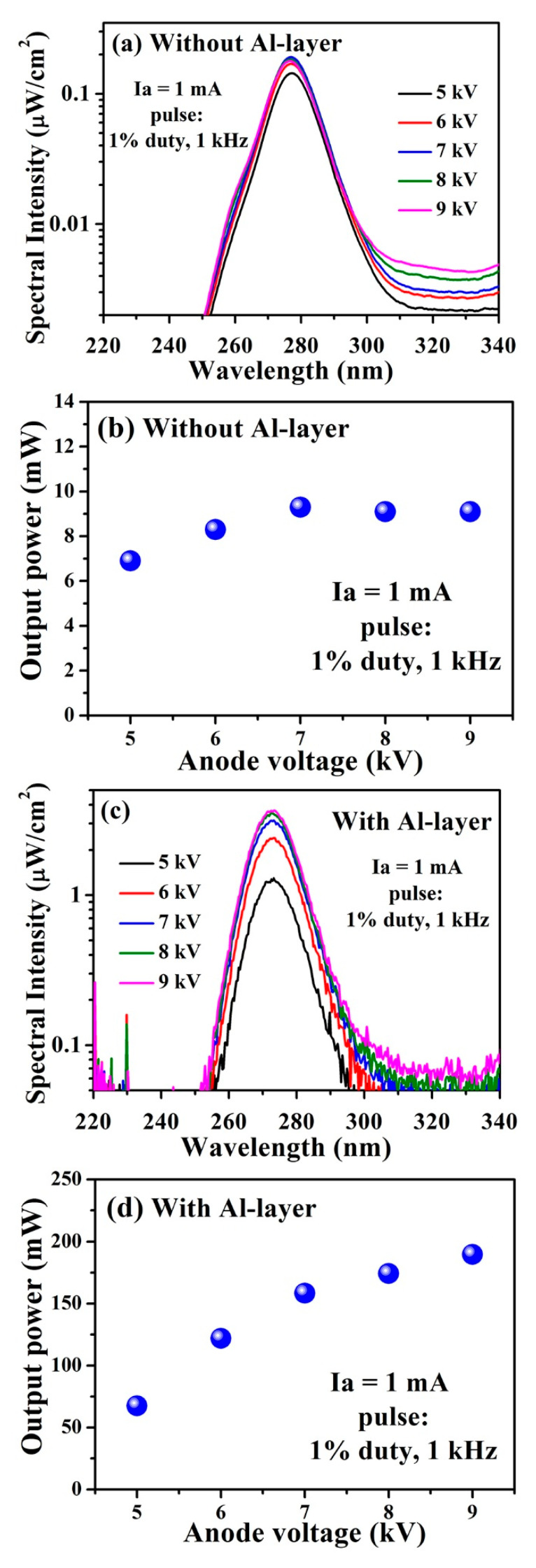
Semi-logarithmic plot of the CL spectra and corresponding measured output power of the UVC–CNTs light source tube-based AlGaN MQWs structure (**a**,**b**) without Al reflector, and (**c**,**d**) with Al reflector, respectively, under various values of the V_A_ at a fixed pulsed-beam current of 1 mA.

To experimentally demonstrate the viability of the Al reflector, an AlGaN-epitaxial structure-based 275 nm UVC emission was fabricated without and with the optimized Al reflector layer of 100 nm to serve as a target (anode) in the UVC–CNTs light source tube, as in Figure 8a. Prior to measuring the performance of the UVC–CNTs light source tube, the selection of an appropriate acceleration voltage (VA) value that matches both AlGaN-based UVC structures with and without the Al reflector is critical. Thus, various V_A_ values in the range (5–9) kV were applied to both structures under a 1 mA fixed anode current (I_A_) and an optimized duty ratio of 1%, with a 1 kHz repetition frequency to minimize the heating effects. The CL spectra of both AlGaN-based UVC structures without and with the Al reflector can be found in Figure 2a,c, respectively. For more qualitative comparison, the CL intensity for all V_A_ values was plotted on a semi-logarithmic scale. Maximal CL intensity and the corresponding output power (Figure 2b) for the AlGaN-based UVC structure without the Al reflector is achieved at V_A_ = 7 kV. These maxima CL intensity and output power values then gradually decrease with increasing V_A_ above 7 kV. In contrast, the AlGaN-based UVC structure with the Al reflector reached the maximal CL intensity and corresponding output power (Figure 2d) at V_A_ = 9 kV. The reason for this difference is the thickness of the Al reflector layer of the AlGaN-based UVC structure. The penetration depth of the incident electron beam on the AlGaN-based UVC structure with the Al reflector is less than that of the AlGaN-based UVC structure without the Al reflector at the same V_A_. 

**Figure 3:**
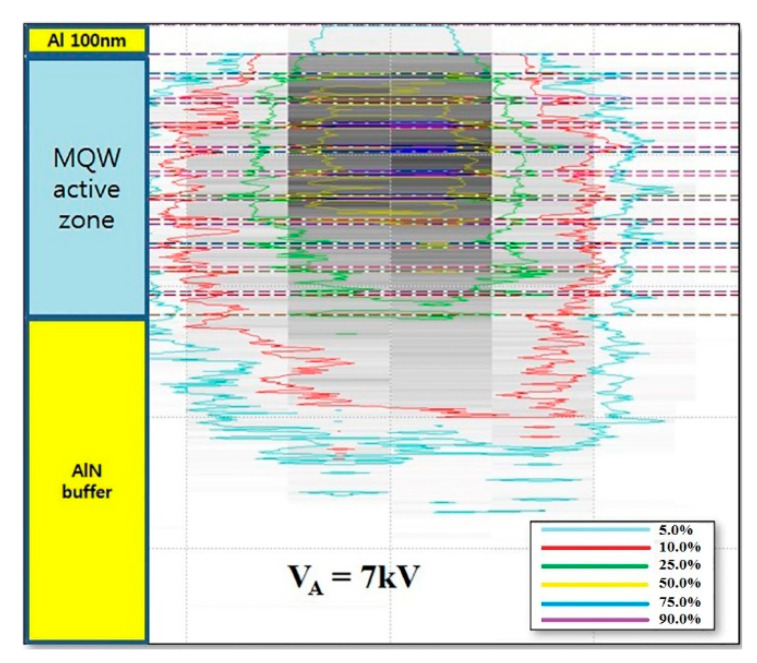
Monte Carlo simulations of the interaction volume for an e-beam energy of 7 kV for AlGaN heterostructure with the optimized Al reflector layer.

Figure 3 shows the Monte Carlo simulation of the interaction volume for an incident electron beam with an AlGaN-based UVC structure with the optimized Al reflector layer of 100 nm in thickness at V_A_ = 7 kV. The simulation model consists of the AlGaN-epitaxial structure mentioned above. For direct comparison, V_A_ = 7 kV is considered as the most appropriate V_A_ in both structures to collect the CL spectra at a range of I_A_.

**Figure 4:**
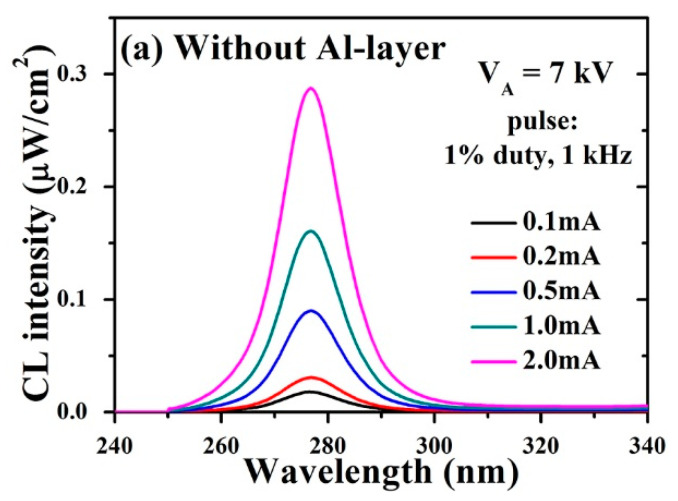

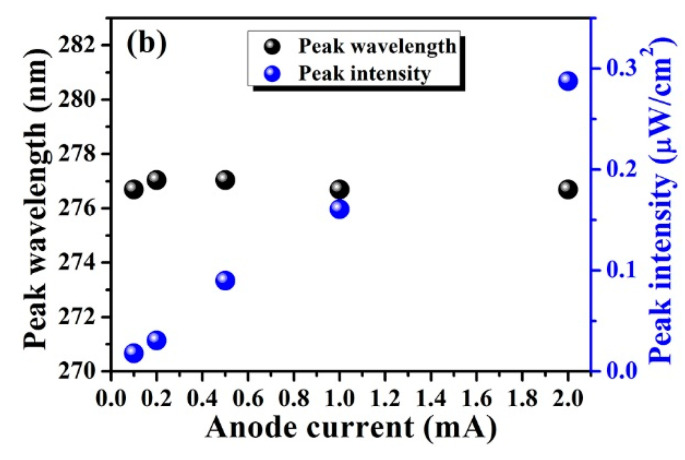
Optical analysis of the UVC–CNTs light source tube-based AlGaN MQWs structure grown without the optimized Al reflector at a fixed accelerating voltage V_A_ of 7 kV (**a**) Dependence of the CL spectra on the anode current I_A_; (**b**) peak wavelength and peak intensity of the CL spectra in (**a**).

After selecting the appropriate V_A_ for both structures, the CL spectra were recorded over a range of pulsed-beam currents with 1% duty ratio and 1 kHz repetition frequency. Figure 4a shows the CL spectra collected from the fabricated UVC–CNTs light source tube-based AlGaN structure without the Al reflector. On increasing the value of I_A_ up to 2 mA, the CL intensity was linearly increased with a peak CL intensity of ~0.29 μW/cm^2^, and the wavelength emission peaked at ~276 nm. Furthermore, no defect luminescence was observed in the UV spectral range. This observation was substantiated through the semi-logarithmic plot of CL intensity at different values of V_A_ in Figure 2a. Figure 4b shows more details, with only slight variations in the peak emission wavelength, which was within the error range of about ±0.3 nm.

**Figure 5:**
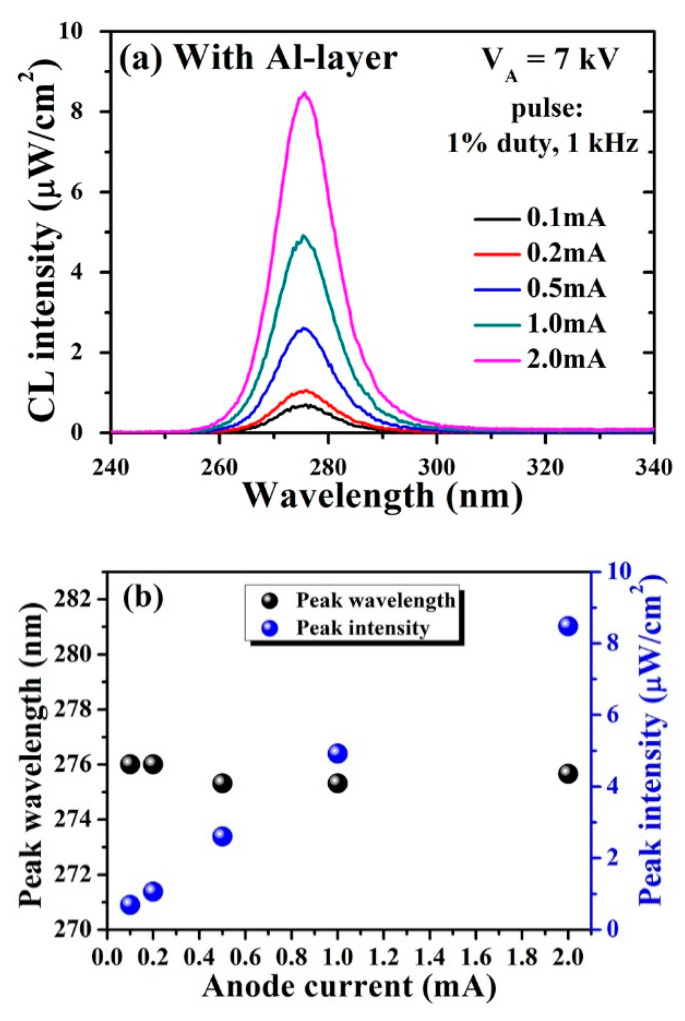
Optical analysis of the UVC–CNTs light source tube-based AlGaN MQWs structure with the optimized Al reflector at a fixed V_A_ of 7 kV. (**a**) Dependence of the CL spectra on the anode current I_A_; (**b**) peak wavelength and peak intensity of the CL spectra in (**a**).

Figure 5a shows the CL spectra of the fabricated UVC–CNTs light source tube-based AlGaN structure with the Al reflector under the same pulsed conditions. As the I_A_ varies from (0.1 to 2) mA, the CL intensity gradually increases, since more electrons reach the emission region. Figure 5b shows that the highest value of CL emission intensity of ~8.5 µW/cm^2^ was achieved at I_A_ = 2 mA with an almost unchanged peak wavelength. Importantly, the CL intensity of the fabricated UVC–CNTs light source tube-based AlGaN structure with the Al reflector is approximately 29 times higher than that of the reference at the same I_A_ of 2 mA. The achieved light extraction enhancement for the fabricated UVC–CNTs light source tube-based AlGaN structure with Al reflector layer is mainly attributed to the significant reflection of the Al reflector and improves the light emission in the normal direction.

**Figure 6:**
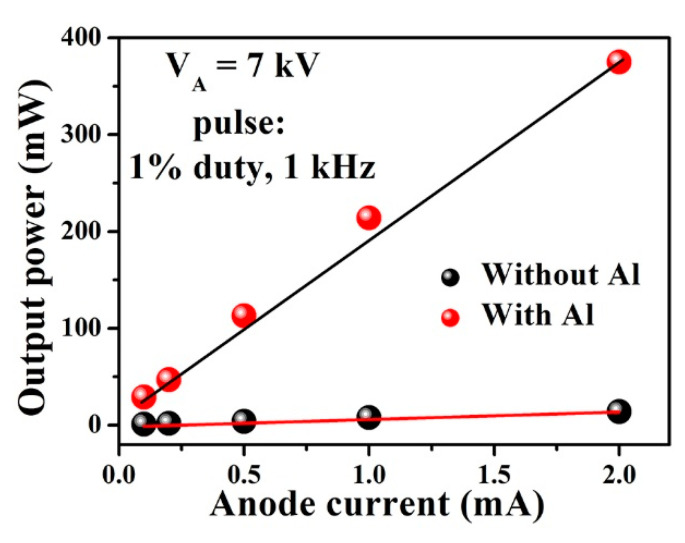
Dependence of the output power of the fabricated UVC–CNTs light source tube-based-AlGaN MQWs structure with and without the optimized Al reflector layer on the anode current I_A_ at a fixed accelerating voltage V_A_ of 7 kV, a duty ratio of 1%, and a repetition frequency of 1 kHz.

Figure 6 shows the measured output power (P_out_) of the UVC–CNTs light source tube-based AlGaN structure without and with the Al reflector under different values of I_A_ at a fixed V_A_ of 7 kV. As the I_A_ increased from (0.1 to 2) mA, the values of P_out_ in both structures were linearly increased without saturation, which indicates that the Pout can be improved further by additionally increasing the I_A_ value. Furthermore, the values of P_out_ of the fabricated UVC–CNTs light source tube-based AlGaN structure with Al reflector are always higher than that of the fabricated UVC–CNTs light source tube-based AlGaN structure without Al reflector over the whole range of I_A_. 

At I_A_ of 2 mA, the UVC–CNTs light source tube-based AlGaN structure with Al reflector achieves the P_out_ value of 375 mW, which is approximately 27 times higher than that of the UVC–CNTs light source tube-based AlGaN structure without an Al reflector of 14 mW. Additionally, the corresponding power efficiency (PE) values for both structures were estimated at an I_A_ of 2 mA and a V_A_ of 7 kV through the given equation, PE = P_out_/(I_A_V_A_). The calculated PE values of the fabricated UVC–CNTs light source tube-based AlGaN structure without and with Al reflector were about (0.1 and 2.68)%, respectively. The results indicate that the PE can be enhanced by applying the Al reflector layer with the optimized thickness on top of the AlGaN MQW structure. The Al reflector layer is commonly employed in GaN-based lighting devices to improve the light extraction efficiency [28,29].

## 3. Materials and Methods

### 3.1. AlGaN Heterostructure Fabrication

**Figure 7:**
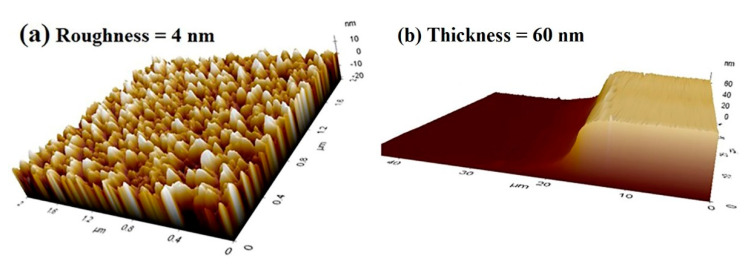
AFM images of one of the Al wafers: (**a**) surface morphology of aluminum with area scan of 2 µm × 2 µm with a surface roughness of 4 nm; (**b**) step-height thickness of aluminum layer measuring ~60 nm.

A combination of hydride vapor phase epitaxy (HVPE) and metal–organic chemical vapor deposition (MOCVD) techniques were utilized to fabricate the AlGaN template-based cathodoluminescence layer on a conventional 2-inch c-plane sapphire substrate for the UVC emission. Firstly, a high-quality 4 μm thick AlN template was fabricated on sapphire substrate at a temperature of 1300 °C by HVPE technique using trimethylaluminum (TMA) and ammonia (NH_3_) precursors. Then, u-AlGaN layer of 1 μm thick was deposited on the AlN template via the MOCVD technique. This was followed by the fabrication of 1 µm thick n-AlGaN layer at a temperature of 1000 °C by supplying TMA, trimethylgallium (TMG), and NH_3_. After that, AlGaN MQWs active region of ~135 nm thick was deposited. The AlGaN/GaN MQWs active region consisted of 10 pairs of a 2.5 nm thick AlGaN quantum wells layer with an aluminum (Al) composition of 45%, and a 10 nm thick GaN quantum barrier. This was followed by a coating of an Al cap layer with different thicknesses on the surface of the AlGaN template as a reflector layer using an electron beam evaporator technique followed by rapid thermal annealing at 250 °C for 20 s to improve the light extraction by reflecting backscattering UV emission. Figure 7 shows atomic force microscopy (AFM) images of one of the Al wafers to understand the surface morphology (Figure 7a) and to confirm the deposited thickness of the Al thin film (Figure 7b).

### 3.2. Carbon Nanotubes Emitter Preparation 

A simple screen-printing process was employed to fabricate the field emitter-based cathode with 300 μm diameter dots of the desired arrays of carbon nanotubes (CNTs) to use as an electron emission source. Firstly, well-dispersed CNTs paste was printed on the top surface of 2-inch stainless steel 316 (SS316). Then, all residual organic materials in the SS316 substrate with CNTs dots emitter were eliminated by a heat-treating process in a vacuum ambient at high temperature. After that, to obtain nanotubes with vertical alignment prior to use in field emission devices as an emitter, a physical activation process was made to the surface of the heat-treated CNT dots emitter using an adhesive tape and soft roller. For a more stable emission current, the surface-activated CNT field emitter was followed by an aging process. This process was performed to enhance the emission uniformity and lifetime of the crooked and long CNTs by applying a high electric field for a long time at different levels of emission current, prior to serving as a stable field emission cathode in the well-sealed UVC–CNTs light source tube. In a high vacuum chamber, various levels of DC emission current starting from (0.1 to 1) mA were applied to the CNTs emitter on SS316 substrate, which make them stand straight and parallel along with fields with approximately the same height. More details of the preparation process of CNTs field emitters were cited in our previous works [26,30]. 

### 3.3. Design of the UVC–CNTs Device and CL Measurements

**Figure 8:**
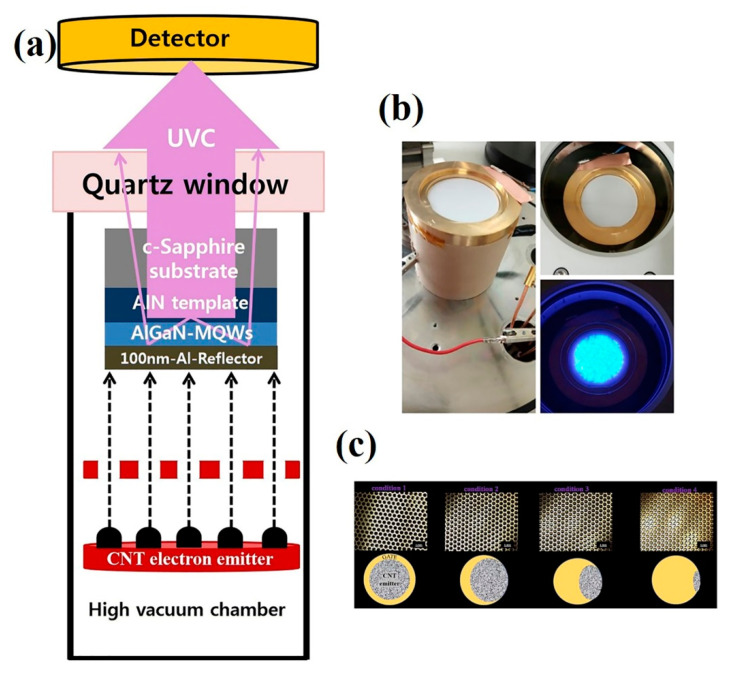
(**a**) Schematic of the well-sealed UVC–CNTs light source tube in a high vacuum chamber; (**b**) photographs of UVC-CNTs light source tube prototype; and (**c**) gate to CNTs emitter alignment with four conditions.

The CNTs dots emitter on SS316 substrate as a cathode, the AlGaN template as an anode, and a metal mesh gate electrode were collected and sealed together with insulator spacers to form the 2-inch UVC–CNTs light source tube with triode-type structure. Prior to loading the well-sealed UVC–CNTs light source tube in a high vacuum chamber covered with a UV-transparent quartz window to detect the CL spectra, the CNTs emitter dots and the metal mesh gate electrode holes were well-aligned to improve the transmission of the emission current from the cathode to the anode as shown in Figure 8c. The well-aligned UVC–CNTs light source tube was centered in the vacuum chamber close to the quartz exit window. The turbomolecular pump and dry vacuum roughing pump machines were used to evacuate this vacuum chamber. 

To emit the electrons from the CNTs-based cathode, the anode and gate electrode were connected to a positive DC voltage, and to square high voltage pulses with various duty ratios and frequencies, respectively. The emitted electrons from the CNTs cathode excited the AlGaN/GaN MQWs, causing the MQW layers to emit UVC emission. A calibrated photo-detector fitted with an optical fiber was utilized to detect the UVC emission. To record the CL emission spectra and their output power, a high-sensitivity spectrometer (Maya2000 Pro) was centered in close proximity to the quartz window. Figure 8a,b show a schematic of the well-sealed UVC–CNTs light source tube in a high vacuum chamber and photographs of UVC-CNTs light source tube prototype, respectively. 

## 4. Conclusions

In summary, an optimized Al reflector layer was proposed and demonstrated for the light extraction efficiency enhancement of the mass scale UVC–CNTs light source tube-based AlGaN MQW structure. The thickness of the Al reflector layer was optimized by optical measurements of UVC-CNTs light source tube. The results demonstrate that the highest enhancement of the light extraction efficiency of UVC–CNTs light source tube structure was achieved when the Al reflector possessed a thickness of 100 nm. Then, the optimized Al reflector was fabricated and applied in the UVC–CNTs light source tube-based AlGaN MQW structure. For comparison, the CL spectra for the UVC–CNTs light source tube-based AlGaN MQW structure with and without the optimized Al reflector were recorded at an accelerating voltage (V_A_) of 7 kV, which was the most suitable V_A_ for both structures. The intensity of CL emission was reliant on the value of anode current (I_A_) in both structures. At I_A_ of 2 mA, the output power of the UVC–CNTs light source tube-based AlGaN MQW structure with the optimized Al reflector was ~ 27 times higher than that of the reference UVC–CNTs light source tube without the optimized Al reflector. The corresponding power efficiency for the well-sealed UVC–CNTs light source tube-based AlGaN MQWs structure with the optimized Al reflector was 2.68%, compared to only 0.1% of the reference UVC–CNTs light source tube without the optimized Al reflector. This study provides an efficient method to realize the high-power efficiency UVC–CNTs light source tubes for potential applications.

## Data Availability

Data is contained within the article.

## Sample Availability

Samples of the compounds are not available from the authors.
